# Supplementary material for: Spatially Explicit Modeling of Anthropogenic Heat Intensity in Beijing Center Area: An Investigation of Driving Factors with Urban Spatial Forms
Source: Sensors (Basel). 2023 Sep 1;23(17):7608. doi: 10.3390/s23177608 (PMC10490750; doi:10.3390/s23177608)
Supplement: Supplementary file 1 [file sensors-23-07608-s001.zip › sensors-2500151-supplementary.pdf]

Article

Spatially Explicit Modeling of Anthropogenic Heat Intensity in Beijing Center Area: An Investigation of Driving Factors with Urban Spatial Forms

Meizi Yang [1,2], Shisong Cao [2] and Dayu Zhang [1,*]

[1] School of Architecture and Urban Planning, Beijing University of Civil Engineering and Architecture, Beijing 100044, China
[2] School of Geomatics and Urban Spatial Informatics, Beijing University of Civil Engineering and Architecture, Beijing 100044, China.
* Correspondence: zhangdy@bucea.edu.cn

Table S1. Correlation analysis of AHF and urban form elements in functional zone A.

		AHF	BD	BH	FAR	BV	FC	FD	FVC	AIA
AHF	Pearson Correlation	1	0.098**	0.293**	0.339**	0.228**	0.032	0.188**	−0.267**	0.241**
	Sig. (2-tailed)		0.007	0.000	0.000	0.000	0.374	0.000	0.000	0.000
	N	764	764	764	764	764	764	764	764	764
BD	Pearson Correlation	0.098**	1	−0.130**	0.547**	−0.059	0.019	0.298**	−0.621**	0.531**
	Sig. (2-tailed)	0.007		0.000	0.000	0.105	0.597	0.000	0.000	0.000
	N	764	764	764	764	764	764	764	764	764
BH	Pearson Correlation	0.293**	−0.130**	1	0.563**	0.605**	−0.019	0.097**	−0.054	0.065
	Sig. (2-tailed)	0.000	0.000		0.000	0.000	0.601	0.007	0.137	0.072
	N	764	764	764	764	764	764	764	764	764
FAR	Pearson Correlation	0.339**	0.547**	0.563**	1	0.467**	−0.008	0.360**	−0.492**	0.488**
	Sig. (2-tailed)	0.000	0.000	0.000		0.000	0.818	0.000	0.000	0.000

	N	764	764	764	764	764	764	764	764	764
**BV**	Pearson Correlation	0.228**	−0.059	0.605**	0.467**	1	−0.066	0.000	−0.027	−0.126**
	Sig. (2-tailed)	0.000	0.000	0.000	0.000		0.611	0.004	0.504	0.000
	N	719	719	719	719	719	719	719	719	719
**FC**	Pearson Correlation	0.009	0.198**	0.022	0.128**	0.019	1	−0.326**	−0.193**	0.200**
	Sig. (2-tailed)	0.818	0.000	0.557	0.001	0.611		0.000	0.000	0.000
	N	719	719	719	719	719	719	719	719	719
**FD**	Pearson Correlation	0.172**	0.196**	0.140**	0.291**	0.108**	−0.326**	1	−0.149**	0.260**
	Sig. (2-tailed)	0.000	0.000	0.000	0.000	0.004	0.000		0.000	0.000
	N	719	719	719	719	719	719	719	719	719
**FVC**	Pearson Correlation	−0.110**	−0.588**	−0.001	−0.386**	−0.025	−0.193**	−0.149**	1	−0.606**
	Sig. (2-tailed)	0.003	0.000	0.979	0.000	0.504	0.000	0.000		0.000
	N	719	719	719	719	719	719	719	719	719
**AIA**	Pearson Correlation	0.089*	0.547**	0.023	0.363**	−0.162**	0.200**	0.260**	−0.606**	1
	Sig. (2-tailed)	0.017	0.000	0.540	0.000	0.000	0.000	0.000	0.000	
	N	719	719	719	719	719	719	719	719	719

**. Correlation is significant at the 0.01 level (2-tailed).

Table S2. Correlation analysis of AHF and urban form elements in functional zone B.

		AHF	BD	BH	FAR	BV	FC	FD	FVC	AIA
**AHF**	Pearson Correlation	1	0.148**	0.360**	0.498**	0.223**	0.033	0.376**	−0.210**	0.293**
	Sig. (2-tailed)		0.000	0.000	0.000	0.000	0.287	0.000	0.000	0.000
	N	1072	1072	1072	1072	1072	1072	1072	1072	1072
**BD**	Pearson Correlation	0.148**	1	−0.013	0.558**	0.063*	0.183**	0.178**	−0.445**	0.439**
	Sig. (2-tailed)	0.000		0.664	0.000	0.040	0.000	0.000	0.000	0.000
	N	1072	1072	1072	1072	1072	1072	1072	1072	1072
**BH**	Pearson Correlation	0.360**	−0.013	1	0.608**	0.442**	0.072*	0.371**	−0.131**	0.244**
	Sig. (2-tailed)	0.000	0.664		0.000	0.000	0.019	0.000	0.000	0.000
	N	1072	1072	1072	1072	1072	1072	1072	1072	1072
**FAR**	Pearson Correlation	0.498**	0.558**	0.608**	1	0.400**	0.204**	0.414**	−0.363**	0.484**
	Sig. (2-tailed)	0.000	0.000	0.000		0.000	0.000	0.000	0.000	0.000
	N	1072	1072	1072	1072	1072	1072	1072	1072	1072
**BV**	Pearson Correlation	0.223**	0.063*	0.442**	0.400**	1	0.020	0.055	−0.095**	−0.068*
	Sig. (2-tailed)	0.000	0.040	0.000	0.000		0.512	0.071	0.002	0.026
	N	1072	1072	1072	1072	1072	1072	1072	1072	1072
**FC**	Pearson Correlation	0.033	0.183**	0.072*	0.204**	0.020	1	−0.257**	−0.222**	0.225**

	Sig. (2-tailed)	.287	0.000	0.019	0.000	0.512		0.000	0.000	0.000
	N	1072	1072	1072	1072	1072	1072	1072	1072	1072
**FD**	Pearson Correlation	0.376**	0.178**	0.371**	0.414**	0.055	−0.257**	1	−0.163**	0.389**
	Sig. (2-tailed)	0.000	0.000	0.000	0.000	0.071	0.000		0.000	0.000
	N	1072	1072	1072	1072	1072	1072	1072	1072	1072
**FVC**	Pearson Correlation	−0.210**	−0.445**	−0.131**	−0.363**	−0.095**	−0.222**	−0.163**	1	−0.497**
	Sig. (2-tailed)	0.000	0.000	0.000	0.000	0.002	0.000	0.000		0.000
	N	1072	1072	1072	1072	1072	1072	1072	1072	1072
**AIA**	Pearson Correlation	0.293**	0.439**	0.244**	0.484**	−0.068*	0.225**	0.389**	−0.497**	1
	Sig. (2-tailed)	0.000	0.000	0.000	0.000	0.026	0.000	0.000	0.000	
	N	1072	1072	1072	1072	1072	1072	1072	1072	1072

**. Correlation is significant at the 0.01 level (2-tailed).

Table S3. Correlation analysis of AHF and urban form elements in functional zone R.

		**AHF**	**BD**	**BH**	**FAR**	**BV**	**FC**	**FD**	**FVC**	**AIA**
**AHF**	Pearson Correlation	1	−0.124**	0.185**	0.188**	0.276**	0.009	0.172**	−0.110**	0.089*
	Sig. (2-tailed)		0.001	0.000	0.000	0.000	0.818	0.000	0.003	0.017
	N	719	719	719	719	719	719	719	719	719
**BD**	Pearson Correlation	−0.124**	1	−0.170**	0.453**	−0.148**	0.198**	0.196**	−0.588**	0.547**
	Sig. (2-tailed)	0.001		0.000	0.000	0.000	0.000	0.000	0.000	0.000

	N	719	719	719	719	719	719	719	719	719
**BH**	Pearson Correlation	0.185**	−0.170**	1	0.647**	0.687**	0.022	0.140**	−0.001	0.023
	Sig. (2-tailed)	0.000	0.000		0.000	0.000	0.557	0.000	0.979	0.540
	N	719	719	719	719	719	719	719	719	719
**FAR**	Pearson Correlation	0.188**	0.453**	0.647**	1	0.594**	0.128**	0.291**	−0.386**	0.363**
	Sig. (2-tailed)	0.000	0.000	0.000		0.000	0.001	0.000	0.000	0.000
	N	719	719	719	719	719	719	719	719	719
**BV**	Pearson Correlation	0.276**	−0.148**	0.687**	0.594**	1	0.019	0.108**	−0.025	−0.162**
	Sig. (2-tailed)	0.000	0.000	0.000	0.000		0.611	0.004	0.504	0.000
	N	719	719	719	719	719	719	719	719	719
**FC**	Pearson Correlation	0.009	0.198**	0.022	0.128**	0.019	1	−0.326**	−0.193**	0.200**
	Sig. (2-tailed)	0.818	0.000	0.557	0.001	0.611		0.000	0.000	0.000
	N	719	719	719	719	719	719	719	719	719
**FD**	Pearson Correlation	0.172**	0.196**	0.140**	0.291**	0.108**	−0.326**	1	−0.149**	0.260**
	Sig. (2-tailed)	0.000	0.000	0.000	0.000	0.004	0.000		0.000	0.000
	N	719	719	719	719	719	719	719	719	719
**FVC**	Pearson Correlation	−0.110**	−0.588**	−0.001	−0.386**	−0.025	−0.193**	−0.149**	1	−0.606**
	Sig. (2-tailed)	0.003	0.000	0.979	0.000	0.504	0.000	0.000		0.000

	N	719	719	719	719	719	719	719	719	719
**AIA**	Pearson Correlation	0.089*	0.547**	0.023	0.363**	−0.162**	0.200**	0.260**	−0.606**	1
	Sig. (2-tailed)	0.017	0.000	0.540	0.000	0.000	0.000	0.000	0.000	
	N	719	719	719	719	719	719	719	719	719

**. Correlation is significant at the 0.01 level (2-tailed).

*. Correlation is significant at the 0.05 level (2-tailed).

Table S4. Correlation analysis of AHF and urban form elements in functional zone G.

		**AHF**	**BD**	**BH**	**FAR**	**BV**	**FC**	**FD**	**FVC**	**AIA**
**AHF**	Pearson Correlation	1	0.424**	0.354**	0.428**	0.134**	0.340**	0.288**	−0.522**	0.537**
	Sig. (2-tailed)		0.000	0.000	0.000	0.000	0.000	0.000	0.000	0.000
	N	1798	1798	1798	1798	1798	1798	1798	1798	1798
**BD**	Pearson Correlation	0.424**	1	0.298**	0.846**	0.106**	0.323**	0.330**	−0.526**	0.636**
	Sig. (2-tailed)	0.000		0.000	0.000	0.000	0.000	0.000	0.000	0.000
	N	1798	1798	1798	1798	1798	1798	1798	1798	1798
**BH**	Pearson Correlation	0.354**	0.298**	1	0.500**	0.585**	0.233**	0.102**	−0.349**	0.350**
	Sig. (2-tailed)	0.000	0.000		0.000	0.000	0.000	0.000	0.000	0.000
	N	1798	1798	1798	1798	1798	1798	1798	1798	1798
**FAR**	Pearson Correlation	0.428**	0.846**	0.500**	1	0.257**	0.280**	0.305**	−0.493**	0.556**
	Sig. (2-tailed)	0.000	0.000	0.000		0.000	0.000	0.000	0.000	0.000

	N	1798	1798	1798	1798	1798	1798	1798	1798	1798
**BV**	Pearson Correlation	0.134**	0.106**	0.585**	0.257**	1	0.039	0.015	−0.157**	0.131**
	Sig. (2-tailed)	0.000	0.000	0.000	0.000		0.095	0.522	0.000	0.000
	N	1798	1798	1798	1798	1798	1798	1798	1798	1798
**FC**	Pearson Correlation	0.340**	0.323**	0.233**	0.280**	0.039	1	0.034	−0.380**	0.394**
	Sig. (2-tailed)	0.000	0.000	0.000	0.000	0.095		0.147	0.000	0.000
	N	1798	1798	1798	1798	1798	1798	1798	1798	1798
**FD**	Pearson Correlation	0.288**	0.330**	0.102**	0.305**	0.015	0.034	1	−0.250**	0.282**
	Sig. (2-tailed)	0.000	0.000	0.000	0.000	0.522	0.147		0.000	0.000
	N	1798	1798	1798	1798	1798	1798	1798	1798	1798
**FVC**	Pearson Correlation	−0.522**	−0.526**	−0.349**	−0.493**	−0.157**	−0.380**	−0.250**	1	−0.756**
	Sig. (2-tailed)	0.000	0.000	0.000	0.000	0.000	0.000	0.000		0.000
	N	1798	1798	1798	1798	1798	1798	1798	1798	1798
**AIA**	Pearson Correlation	0.537**	0.636**	0.350**	0.556**	0.131**	0.394**	0.282**	−0.756**	1
	Sig. (2-tailed)	0.000	0.000	0.000	0.000	0.000	0.000	0.000	0.000	
	N	1798	1798	1798	1798	1798	1798	1798	1798	1798

**. Correlation is significant at the 0.01 level (2-tailed).

*. Correlation is significant at the 0.05 level (2-tailed).

Table S5. Correlation analysis of AHF and urban form elements in functional zone M.

		AHF	BD	BH	FAR	BV	FC	FD	FVC	AIA
**AHF**	Pearson Correlation	1	0.329**	0.227**	0.354**	0.145**	0.157**	0.199**	−0.309**	0.304**
	Sig. (2-tailed)		0.000	0.000	0.000	0.000	0.000	0.000	0.000	0.000
	N	755	755	755	755	755	755	755	755	755
**BD**	Pearson Correlation	0.329**	1	0.255**	0.863**	0.134**	0.214**	0.323**	−0.418**	0.505**
	Sig. (2-tailed)	0.000		0.000	0.000	0.000	0.000	0.000	0.000	0.000
	N	755	755	755	755	755	755	755	755	755
**BH**	Pearson Correlation	0.227**	0.255**	1	0.367**	0.517**	0.137**	0.180**	−0.167**	0.250**
	Sig. (2-tailed)	0.000	0.000		0.000	0.000	0.000	0.000	0.000	0.000
	N	755	755	755	755	755	755	755	755	755
**FAR**	Pearson Correlation	0.354**	0.863**	0.367**	1	0.251**	0.116**	0.350**	−0.367**	0.450**
	Sig. (2-tailed)	0.000	0.000	0.000		0.000	0.001	0.000	0.000	0.000
	N	755	755	755	755	755	755	755	755	755
**BV**	Pearson Correlation	0.145**	0.134**	0.517**	0.251**	1	−0.040	0.032	−0.086*	0.060
	Sig. (2-tailed)	0.000	0.000	0.000	0.000		0.276	0.378	0.018	0.098
	N	755	755	755	755	755	755	755	755	755
**FC**	Pearson Correlation	0.157**	0.214**	0.137**	0.116**	−0.040	1	−0.014	−0.174**	0.230**

	Sig. (2-tailed)	0.000	0.000	0.000	0.001	0.276		0.704	0.000	0.000
	N	755	755	755	755	755	755	755	755	755
**FD**	Pearson Correlation	0.199**	0.323**	0.180**	0.350**	0.032	−0.014	1	−0.260**	0.278**
	Sig. (2-tailed)	0.000	0.000	0.000	0.000	0.378	0.704		0.000	0.000
	N	755	755	755	755	755	755	755	755	755
**FVC**	Pearson Correlation	−0.309**	−0.418**	−0.167**	−0.367**	−0.086*	−0.174**	−0.260**	1	−0.567**
	Sig. (2-tailed)	0.000	0.000	0.000	0.000	0.018	0.000	0.000		0.000
	N	755	755	755	755	755	755	755	755	755
**AIA**	Pearson Correlation	0.304**	0.505**	0.250**	0.450**	0.060	0.230**	0.278**	−0.567**	1
	Sig. (2-tailed)	0.000	0.000	0.000	0.000	0.098	0.000	0.000	0.000	
	N	755	755	755	755	755	755	755	755	755

**. Correlation is significant at the 0.01 level (2-tailed).

*. Correlation is significant at the 0.05 level (2-tailed).

Table S6. Correlation analysis of AHF and urban form elements in functional zone S.

		AHF	BD	BH	FAR	BV	FC	FD	FVC	AIA
**AHF**	Pearson Correlation	1	0.528**	0.009	0.567**	0.625**	−0.409**	0.023	−0.783**	0.589**
	Sig. (2-tailed)		0.000	0.940	0.000	0.000	0.000	0.851	0.000	0.000
	N	72	72	72	72	72	72	72	72	72
**BD**	Pearson Correlation	0.528**	1	0.136	0.903**	0.361**	−0.109	0.251*	−0.457**	0.488**

	Sig. (2-tailed)	0.000		0.254	0.000	0.002	0.363	0.033	0.000	0.000
	N	72	72	72	72	72	72	72	72	72
	Pearson Correlation	0.009	0.136	1	0.356**	0.195	0.219	0.467**	−0.167	0.256*
**BH**	Sig. (2-tailed)	0.940	0.254		0.002	0.101	0.064	0.000	0.160	0.030
	N	72	72	72	72	72	72	72	72	72
	Pearson Correlation	0.567**	0.903**	0.356**	1	0.458**	−0.109	0.520**	−0.498**	0.490**
**FAR**	Sig. (2-tailed)	0.000	0.000	0.002		0.000	0.363	0.000	0.000	0.000
	N	72	72	72	72	72	72	72	72	72
	Pearson Correlation	0.625**	0.361**	0.195	0.458**	1	−0.185	0.041	−0.377**	0.275*
**BV**	Sig. (2-tailed)	0.000	0.002	0.101	0.000		0.119	0.735	0.001	0.020
	N	72	72	72	72	72	72	72	72	72
	Pearson Correlation	−0.409**	−0.109	0.219	−0.109	−0.185	1	0.081	0.369**	−0.199
**FC**	Sig. (2-tailed)	0.000	0.363	0.064	0.363	0.119		0.500	0.001	0.094
	N	72	72	72	72	72	72	72	72	72
	Pearson Correlation	0.023	0.251*	0.467**	0.520**	0.041	0.081	1	−0.188	0.242*
**FD**	Sig. (2-tailed)	0.851	0.033	0.000	0.000	0.735	0.500		0.115	0.040
	N	72	72	72	72	72	72	72	72	72
	Pearson Correlation	−0.783**	−0.457**	−0.167	−0.498**	−0.377**	0.369**	−0.188	1	−0.738**
**FVC**	Sig. (2-tailed)	0.000	0.000	0.160	0.000	0.001	0.001	0.115		0.000
	N	72	72	72	72	72	72	72	72	72
**AIA**	Pearson Correlation	0.589**	0.488**	0.256*	0.490**	0.275*	−0.199	0.242*	−0.738**	1

Sig. (2-tailed)	0.000	0.000	0.030	0.000	0.020	0.094	0.040	0.000	
N	72	72	72	72	72	72	72	72	72

**. Correlation is significant at the 0.01 level (2-tailed).

*. Correlation is significant at the 0.05 level (2-tailed).

Table S7. Multicollinearity test among urban form elements

	VIF					
	**A**	**B**	**R**	**G**	**M**	**S**
BD	2.877	2.268	3.202	4.692	4.576	5.664
BH	2.644	2.408	3.344	2.120	1.589	-
FAR	4.320	3.619	5.297	4.686	4.585	6.289
BV	1.964	1.470	2.805	1.557	1.439	1.359
FC	-	-	-	1.258	1.136	1.191
FD	1.238	1.349	1.132	1.157	1.201	-
FVC	1.995	1.478	1.961	2.441	1.549	2.709
AIA	2.036	1.850	2.102	2.953	1.767	2.375

Table S8. Regression results and t test of urban form elements in functional zone A.

	**Unstandardized Coefficients**		**Standardized Coefficients**	**t**	**P**	**VIF**	**R^2**	**Adjusted R^2**	**F**
	B	Std. Error	Beta						
(Constant)	0.078	0.013	0	6.074	0.000***	-	0.181	0.173	F=23.835 P=0.000***
BD	−0.063	0.02	−0.171	−3.069	0.002***	2.877			

BH	0.037	0.019	0.105	1.947	0.052*	2.664
FAR	0.076	0.036	0.146	2.137	0.033**	4.32
BV	0.684	0.327	0.096	2.091	0.037**	1.964
FD	0.054	0.028	0.070	1.904	0.057*	1.238
FVC	−0.074	0.017	−0.200	−4.291	0.000***	1.995
AIA	0.028	0.011	0.121	2.573	0.010**	2.036

Dependent variable: AHF

***. Correlation is significant at the 0.01 level (2-tailed). **. Correlation is significant at the 0.05 level (2-tailed). *. Correlation is significant at the 0.1 level (2-tailed).

Table S9. Regression results and t test of urban form elements in functional zone B.

	**Unstandardized Coefficients**		**Standardized Coefficients**	**t**	**P**	**VIF**	**R^2**	**Adjusted R^2**	**F**
	B	Std. Error	Beta						
(Constant)	0.065	0.017	-	3.827	0.000***	-			
BD	−0.152	0.024	−0.243	−6.354	0.000***	2.268			
BH	−0.051	0.024	−0.083	−2.099	0.036**	2.408			
FAR	0.361	0.033	0.532	11.019	0.000***	3.619	0.314	0.310	F=69.658 P=0.000***
BV	0.328	0.21	0.048	1.565	0.118	1.47			
FD	0.157	0.024	0.194	6.591	0.000***	1.349			
FVC	−0.054	0.023	−0.072	−2.334	0.020**	1.478			
AIA	0.026	0.017	0.054	1.57	0.117	1.85			

Dependent variable: AHF

***. Correlation is significant at the 0.01 level (2-tailed). **. Correlation is significant at the 0.05 level (2-tailed). *. Correlation is significant at the 0.1 level (2-tailed).

Table S10. Regression results and t test of urban form elements in functional zone R.

	**Unstandardized Coefficients**		**Standardized Coefficients**	**t**	**P**	**VIF**	**R^2**	**Adjusted R^2**	**F**
	B	Std. Error	Beta						
(Constant)	0.126	0.026	-	4.902	0.000***	-	0.175	0.168	F=21.650 P=0.000***
BD	−0.313	0.043	−0.443	−7.275	0.000***	3.202			
BH	−0.1	0.027	−0.230	−3.692	0.000***	3.344			
FAR	0.167	0.053	0.246	3.141	0.002***	5.297			
BV	2.59	0.643	0.230	4.027	0.000***	2.805			
FD	0.115	0.032	0.131	3.606	0.000***	1.132			
FVC	−0.097	0.03	−0.155	−3.26	0.001***	1.961			
AIA	0.064	0.02	0.157	3.17	0.002***	2.102			

Dependent variable: AHF

***. Correlation is significant at the 0.01 level (2-tailed). **. Correlation is significant at the 0.05 level (2-tailed). *. Correlation is significant at the 0.1 level (2-tailed).

Table S11. Regression results and t test of urban form elements in functional zone G.

	Unstandardized Coefficients		Standardized Coefficients	t	P	VIF	R^2	Adjusted R^2	F
	B	Std. Error	Beta						
(Constant)	0.026	0.003	0	7.732	0.000***	-			
BH	0.033	0.005	0.133	6.092	0.000***	1.374			
FAR	0.042	0.016	0.067	2.680	0.007***	1.798			
FC	0.014	0.002	0.125	6.059	0.000***	1.231	0.377	0.375	F=180.909 P=0.000***
FD	0.106	0.015	0.139	6.982	0.000***	1.146			
AIA	0.024	0.003	0.216	7.053	0.000***	2.689			
FVC	−0.027	0.004	−0.197	−6.767	0.000***	2.439			

Dependent variable: AHF

***. Correlation is significant at the 0.01 level (2-tailed). **. Correlation is significant at the 0.05 level (2-tailed). *. Correlation is significant at the 0.1 level (2-tailed).

Table S12. Regression results and t test of urban form elements in functional zone M.

	Unstandardized Coefficients		Standardized Coefficients	t	P	VIF	R^2	Adjusted R^2	F
	B	Std. Error	Beta						
(Constant)	0.031	0.003	0	9.640	0.000***	-			
BH	0.027	0.01	0.096	2.690	0.007***	1.168	0.178	0.174	F=40.735 P=0.000***
FAR	0.101	0.016	0.239	6.330	0.000***	1.301			

FC	0.008	0.003	0.082	2.436	0.015**	1.045
FVC	−0.031	0.006	−0.191	−5.313	0.000***	1.181

Dependent variable: AHF

***. Correlation is significant at the 0.01 level (2-tailed). **. Correlation is significant at the 0.05 level (2-tailed). *. Correlation is significant at the 0.1 level (2-tailed).

Table S13. Regression results and t test of urban form elements in functional zone S.

	**Unstandardized Coefficients**		**Standardized Coefficients**	**t**	**P**	**VIF**	**R^2**	**Adjusted R^2**	**F**
	B	Std. Error	Beta						
(Constant)	0.181	0.014	0	13.132	0.000***	-			
BV	0.264	0.045	0.385	5.807	0.000***	1.166	0. 740	0.733	F=98.353 P=0.000***
FVC	−0.239	0.025	−0.638	−9.633	0.000***	1.166			

Dependent variable: AHF

***. Correlation is significant at the 0.01 level (2-tailed). **. Correlation is significant at the 0.05 level (2-tailed). *. Correlation is significant at the 0.1 level (2-tailed).
